# Burden of informal care giving to patients with psychoses: A descriptive and methodological study

**DOI:** 10.1177/0020764011427239

**Published:** 2013-03

**Authors:** Lena Flyckt, Anna Löthman, Leif Jörgensen, Anders Rylander, Thomas Koernig

**Affiliations:** 1 Stockholm Centre of Psychiatric Research, Karolinska Institutet, Department of Clinical Neurosciences, Stockholm, Sweden; 2 Northern Stockholm Psychiatry, Section of Psychotic Disorders, Stockholm, Sweden; 3 AstraZeneca Nordic, Södertälje, Sweden; 4 AR Health Economic Consultancy, Nykvarn, Sweden

**Keywords:** Informal care giving, schizophrenia, subjective burden, objective burden, diary method, recall method

## Abstract

**Background:**

There is a lack of studies of the size of burden associated with informal care giving in psychosis.

**Aims:**

To evaluate the objective and subjective burden of informal care giving to patients with psychoses, and to compare a diary and recall method for assessments of objective burden.

**Method:**

Patients and their informal caregivers were recruited from nine Swedish psychiatric outpatient centres. Subjective burden was assessed at inclusion using the CarerQoL and COPE index scales. The objective burden (time and money spent) was assessed by the caregivers daily using diaries over four weeks and by recall at the end of weeks 1 and 2.

**Results:**

One-hundred and seven patients (53% females; mean age 43 ± 11) and 118 informal caregivers (67%; 58 ± 15 years) were recruited. Informal caregivers spent 22.5 hours/week and about 14% of their gross income on care-related activities. The time spent was underestimated by two to 20 hours when assessed by recall than by daily diary records. The most prominent aspects of the subjective burden were mental problems.

**Conclusion:**

Despite a substantial amount of time and money spent on care giving, the informal caregivers perceived the mental aspects of burden as the most troublesome. The informal caregiver burden is considerable and should be taken into account when evaluating effects of health care provided to patients with psychoses.

## Introduction

In many western countries, psychiatric asylums have closed down and the number of hospital beds has gradually been reduced with the intention of integrating patients with psychoses into society. In Stockholm, Sweden, for example, there has been a 35% decrease in the number of hospitalized patients with a diagnosis of schizophrenia between 1993 and 1999 and the number of treatment days per patient has been reduced by 75% (Dalman & Wicks, 2000). This deinstitutionalization has led to an increased burden for the patients’ closest relatives in many countries (Honkonen, Saarinen, & Salokangas, 1999; Nordentoft et al., 2010; Rantanen et al., 2009; Ryu et al., 2006).

Following the in- to outpatient shift, the importance of family psychosocial interventions has been evidenced worldwide, indicating that informal care is important for the overall outcome of patients with psychosis. There is, however, a lack of studies of the size of burden associated with informal care giving in schizophrenia (Dixon, 1999; Pharoah, Mari, Rathbone, & Wong, 2010). Functional and social impairment, together with unpredictable and sometimes risky or hostile behaviour, goes beyond that of most chronic disorders and strains the families of the patients (Ochoa et al., 2008). Yet the family burden of schizophrenia, its social and mental consequences and its specific features are still largely unknown (Awad & Voruganti, 2008).

In most cases, informal care giving is based on a pre-existing personal relationship between the caregiver and the patient and no payment is offered for the time and money spent (van den Berg, Brouwer, & Koopmanschap, 2004). Informal care giving can be split up into two components: the subjective and the objective burden (Hoenig & Hamilton, 1966). The objective burden includes the effects of care on the caregiver’s health status, finances and the time devoted to care, whereas the subjective burden deals with how the informal caregiver perceives the burden of care. The latter may be further split into positive and negative experiences of care giving. Both these aspects are important to assess to get a broad picture of the situation (Andren & Elmstahl, 2005; Balducci et al., 2008; Schwartz & Gidron, 2002). The subjective burden of informal care giving has been documented in many papers but there is a lack of studies of the objective burden (Awad & Voruganti, 2008).

The methods for exploring informal care giving in psychotic disorders vary widely between studies, rendering comparisons difficult. As for the subjective burden, there is a lack of consistency between studies in the choice of scales. In a review of scales used to measure the subjective burden of informal care in mental illness it was found that 26 had acceptable psychometric properties but greater consistency between studies was warranted (Harvey et al., 2008). Scales measuring salutogenic factors such as stress-coping abilities may be used to measure the effects of interventions that promote informal caregivers’ well-being (Szmukler et al., 1996).

As for the objective burden, it is often merged into the category ‘indirect costs’, reducing the possibility for further exploration (Awad & Voruganti, 2008). The money spent on informal care giving is relatively unproblematic to accurately assess. The time spent, however, is most often based on recall, which may yield unstable results as indicated by the wide variation between studies; in a European study it was estimated that family members spent six to nine hours per day on informal care giving (Magliano et al., 1998), whereas in an American study the corresponding figure was about two to three hours (Franks, Muennig, Lubetkin, & Jia, 2006). This disparity is probably attributable to methodological differences. A diary method with a prospective assessment of time and money spent for care giving has been found to provide more reliable results compared to a retrospective recall method in a heterogeneous sample of disorders (van den Berg & Spauwen, 2006). Yet, the objective care-giving burden assessed with a retrospective recall method compared to a prospective daily diary method has so far not been methodologically evaluated in patients with psychoses.

The main aims of the study were to describe and quantify both the objective and subjective burden of the informal care giving to patients with psychotic disorders. A secondary aim was to compare a recall method with a diary method for the assessment of the objective burden.

## Subjects and methods

### Subjects

Patients above the age of 18, suffering or having suffered from a psychotic episode and being in need of continuous long-term antipsychotic medication for functional sychoses were considered for the study. Patients having the following diagnoses/symptoms could be included: schizophrenia, schizophreniform disorder, schizoaffective disorder, brief psychotic disorder, delusional disorder and psychotic disorder not otherwise specified. The diagnoses were assessed from the medical records and confirmed by the psychiatrist responsible for the patient. To be considered for inclusion the patient also had to have at least one informal caregiver. Major exclusion criteria for both patients and informal caregivers were a diagnosis of dementia or significant cognitive impairment making the self-assessments unreliable.

### Recruitment procedures

After the recruitment of around a third of the patients, the procedure was changed from consecutive recruitment when patients had an appointment at the respective clinic to a screening method of listed patients in the respective outpatient clinic. This change was motivated by a low recruitment rate and by the aim of including a wider range of patients, both the frequent visitors and those who visited the clinics less often. As soon as the patient consented to participate and agreed that his/her closest informal caregiver(s) could be contacted, a letter was sent by post or contact was made by phone or in person. The most relevant informal caregiver(s), up to two, was/were then invited together with the patient to the psychiatric clinic for further study information by the study coordinator and psychiatrist involved in the study. The original study, as well as the change in recruitment procedure, was approved by the ethics committee at Karolinska Institutet, Stockholm (Diary number: 2007/1623-31).

### Study design and study assessments

At the first study visit, assessments were made of the subjective burden, socio-demographic data and patient characteristics (Table 1). The informal caregivers were then given a computer (or paper) diary for daily assessments of the objective burden during the following four-week period. The time spent was assessed during the first two weeks and expenses were measured during the whole period. At the end each of the weeks (1–4) the informal caregiver was asked to estimate the time spent during the previous week in care giving. The informal caregivers were instructed to account only for time and money spent as a result of the care recipient’s illness. At the end of the follow-up period the informal caregivers were asked to recall any major expenses during the preceding 11 months before the study started. The treatment given to the patients was unchanged throughout the study.

**Table 1.** Assessments of the objective nad subjective burden of informal care to patients with psychiotic disorders: Instruments used in the study.

Concept addressed/scoring	Name of instrument	Number of items	Reference
*Assessments by the informal caregivers*			
‘Subjective’ burden – positive and negative dimensions (Likert scale anchored by ‘no’ and ‘a lot’)	CarerQoL-7D	7	Brouwer, van Exel, van Gorp, & Redekop (2006)
‘Subjective’ burden – overall situation (score 0 to 10 – higher = smaller burden)	CarerQoL-VAS	1	Brouwer, van Exel, van Gorp, & Redekop (2006)
Subjective’ burden – three subscales: Negative impact (score from 6 to 24 – highest negative impact) Positive impact (score from 5 to 20 – highest positive impact) Quality of support (score from 4 to 16 – best support)	COPE index	15 7 4 4	McKee et al. (2003) Balducci et al. (2008)
Productivity consequences of care giving	WPAI	6	Reilly, Bracco, Ricci, Santoro, & Stevens (2004)
Health status of the informal caregivers – five dimensions	EQ-5D	5	Rabin & de Charro (2001)
Global health status of the informal caregivers (score from 0 to 1 – best)	EQ VAS	1	Rabin & de Charro (2001)
HRQoL of informal caregivers	EQ-5D index	n.a.	Dolan & Roberts (2002)
Time spent and expenses related to informal care giving – objective burden	Diary	n/a	n.a.
*Assessments by the investigators*			
Psychosocial functioning of the patient – overall	GAF	1	American Psychiatric Association (1994)
Symptoms – overall picture	GAF	1	American Psychiatric Association (1994)
Clinically relevant symptoms of the patients (total score from 8 to 56 – higher = more symptoms)	RS-S	8	Opler, Yang, Caleo, & Alberti (2007)

n/a = not applicable

n.a. = not assessed

### Instruments used in the study

The subjective burden was assessed by the CarerQoL and the COPE index. Both these instruments measure the positive and the negative dimensions of the care giving, whereas only the CarerQoL provides a measure of the overall situation: the CarerQoL-VAS. The COPE index provides, in addition, an assessment of how the informal caregiver perceives the support received. The Work Productivity and Impairment Questionnaire (WPAI) assesses the effect on the informal caregivers’ work, productivity and regular daily activities. Functional and symptomatic characteristics of the patients were assessed with the eight-item PANSS remission scale using the Structured Clinical Interview for Symptoms of Remission (SCI-SR). Global functioning was assessed by the Global Assessment of Functioning Scale (GAF). Health status of the informal caregivers was assessed by the EQ-5D (Table 1).

### Statistical methods

Data were analysed using conventional descriptive statistics such as mean, median, standard deviation, range and frequency. SAS version 8.02 was used for the analysis. Comparisons between groups were calculated using *t*-tests and Kruskal-Wallis tests. The statistical significance level was set to *p* < .05.

## Results

One-hundred and seven patients (53% females; mean age 43 ± 11 years) and 118 informal caregivers (67%; 58 ± 15 years) were recruited from nine psychiatric outpatient care centres in Sweden covering both urban and rural areas. Eighty-one patients had a diagnosis of schizophrenia (76%) and the rest had other forms of psychotic disorders (*n* = 26; 24%).

### Caregiver and patient general and socioeconomic characteristics

More than two-thirds of the patients lived either together with the informal caregivers in the same household (25%) or so close that they were within a travelling time of 30 minutes by bus, car or train (Table 2). For the patients, the majority of their income came mainly from a pension or other public transfers, whereas 53% ofthe caregivers relied on paid jobs and 38% on pension as their main income. Income from all sources were added, giving a mean of €2153 for the caregivers and €1205 for the patients. The medians were €1797 and €1282, respectively.

**Table 2.** General and socioeconomic characteristics of patients and their informal caregivers.

	Caregivers (*n* = 118)	Patients (*n* = 107)
**Gender and age**		
Proportion women (%)	67	53
Mean age (range)	58 (17–87)	43 (22–68)
Percentage with age of 65 and above (%)	35	4
**Civil status (% in each category)**		
Not married and living alone	13.6	64.5
Married or living together with another person	65.3	23.4
Divorced or separated	16.1	12.1
Widow/widower	5.0	0
**Living conditions (% in each category)**		
Alone	22.0	63.6
With spouse	62.7	21.5
With parents	3.4	5.6
With relatives	5.1	3.8
With children	6.8	1.8
With paid caregiver	0.0	0.9
Missing data	0.0	2.8
**Present housing conditions (% in each category)**		
Own home	n.a.	86.9
Group living	n.a.	9.4
Treatment home	n.a.	0.9
Homeless	n.a.	0.9
Missing data	n.a.	1.9
**Distance from caregiver to patient (% in each category)**		
Living in the same household	n/a	24.6
Within walking distance	n/a	11.9
Within 10 minutes by car/bus/train	n/a	11.9
Within 30 minutes by car/bus/train	n/a	22.0
Within 60 minutes by car/bus/train	n/a	22.0
More than 60 minutes away by car/bus/train	n/a	7.6
**Employment status (% in each category)**		
Employed or running own enterprise	54	16
Unemployed	0	10
Retired/sick pension	39	51
Sheltered jobs	0	21
Other	7	2
**Income per year (€)**		
Mean estimated total income from different sources (range)	2153 (512–4615)	1205 (512–4615)
**Main source of income (% in each category)**		
Support from public funds	7.6	44.9
Salary	53.4	15.0
Support from family, relatives or other akin persons	0.9	0.9
Pension	38.1	39.3

The applied exchange rate was €1 = SEK9.75

n/a=not applicable

n.a.= not assessed

### Clinical characteristics of the patients

Time since diagnosis ranged from 0 to 41 years with a median of 14. The two mean GAF ratings, function and symptom ratings, were 51.5 (± 11.2) and 51.0 (± 11.3), respectively. The PANSS remission scale showed that 36% of the patients were in remission (*M* = 19.2 ± 6.7) .

### Formal care

Most of the formal care consisted of patient visits to psychiatric outpatient facilities, on average about 30 times per year. The admissions to psychiatric hospitals were few, with an average of once every third year. The patients met psychiatrists close to four times a year and psychologists 2.5 times per year on average. The main health care contacts were with psychiatry nurses and contact persons, and occurred 18 and 12 times per year, respectively. For all types of contacts the median was lower than the mean, indicating that a few patients received most of the health and community care. Antipsychotic medication was prescribed to all patients. About 75% were on second-generation antipsychotics (including clozapine).

### Objective burden

According to the daily diary method, the mean total number of hours per week spent by the informal caregivers in support of the care recipients was 22.5 (Table 3). Half of the total time was stand-by time. The mean expenses per month per caregiver was €293, with the four most expensive categories being grocery, other expenses, rent and clothes. When, at first visit, the caregivers were asked to recall greater periodical expenses (rental fee, etc.) for the preceding 11 months, these amounted to an average of €66 per month corresponding to 22% of the monthly expenses measured by the diary method. The total monthly expenses per month per caregiver elicited by the diary method corresponded to 14% of the mean gross income. The informal caregivers rated their productivity while at work to be reduced by around 18%, meaning that they lost about six work hours per week due to reduced productivity because of their care-giving situation (*M* = 18.2± 23.4; median = 10). The reduced productivity while carrying out daily activities was of similar size (*M* = 20.3 ± 23.4; median = 10).

**Table 3.** Number of hours per week spent by informal caregivers in support of their care recipients – measured by the diary method (*n* = 100).

Variables	Total	Household work	Support in practical and economic work	Contacts with health care	Travel to the care recipient	Other	Time reserved or in a stand-by status for the care recipient
*M*	22.5	5.1	0.6	0.9	1.3	3.4	11.2
SD	35.6	14.1	1.2	1.3	2.1	5.2	29.7
Median	13.0	1.0	0.0	0.8	0.3	1.0	1.0
Range	0.3–187.3	0.0–131.0	0.0–7.3	0.0–7.3	0.0–12.3	0.0–22.5	0.0–168.3

Missing values = 18

### Recall versus diary method

**Figure 1.**
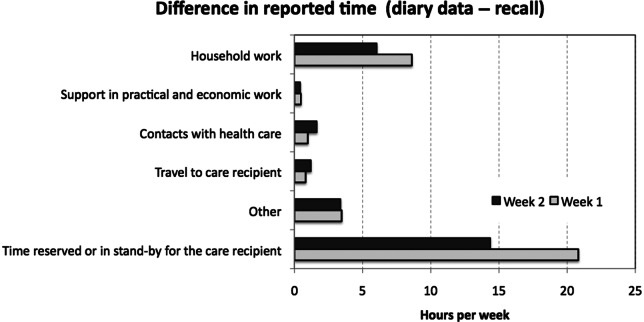
Difference between the diary method and the recall method in assessing the time spent on informal care giving to patients with psychotic disorders. Note: The bars show the difference in time between the diary and recall methods during the first and second week, respectively. For all types of duties, especially the ‘stand-by time’, the time assessed by the diary method exceeded that of the recall method.

The differences between the diary and the recall method during the first and second week of the follow-up period are displayed in Figure 1. For all categories the diary method resulted in higher values. The greatest differences were found for the category ‘stand-by time’ with mean differences of 20 and 15 hours per week during week 1 and 2, respectively. Four explanatory factors were tested for the difference between the recall and diary methods in time spent on care giving; the gender and age (≥ 65 vs < 65 years), if they lived in the same household and the duration of the care recipient’s disease (≥ 15 vs <15 years). These factors were chosen because they were anticipated to explain most of the differences between the methods. There were greater, but not significant, differences between the methods for female compared to male caregivers (*M*: 12.5 ± 29.2 vs. 8.6 ± 17.32, *p* = 0.54).

Elderly caregivers significantly under-reported when using the recall method, while females and persons living in the same household also under-reported, but to a lesser degree. The number of years with the disease had no effect on the reporting method (Table 4).

### Subjective burden – informal caregivers

**Table 4.** Difference between the diary and recall methods in time spent on care giving divided according to the four analysed variables.

Variable	Female	Male	≥ 65	< 65	In the same household	Not in the same household	< 15 years with disease	≥ 15 years with disease
*M*	12.5	8.6	22.8	5.9	18.3	8.6	10.5	10.9
SD	29.2	17.2	39.3	13.7	37.9	19.2	19.6	33.2
Median	3.2	3.2	6.3	2.0	6.2	2.5	3.2	2.5
Range	−24–151	−4–81	−2–151	−24–81	−1–151	−24–117	−4–81	−0.8–151
*p*	0.5389		0.0131		0.5241		0.4904	

*p* from Kruskal-Wallis test

**Figure 2.**
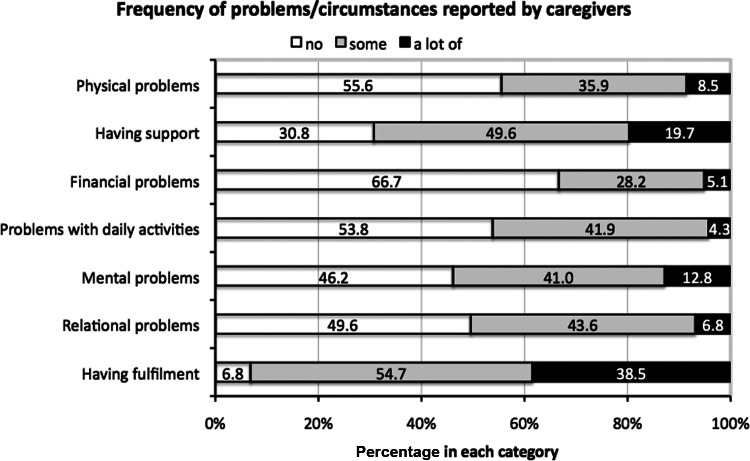
CarerQoL-7D data displaying problems/circumstances linked to the care giving situation.

When using the CarerQoL-7D, around 54% of the informal caregivers reported some or a lot of problems with their own mental health and over 50% reported relational problems with the care recipient (Figure 2). Around a third of caregivers felt that they did not receive any support in their roles. More than 90% reported that they, to some extent, felt fulfilment in their care tasks.

CarerQoL-VAS, a summary measure of the subjective burden encompassing both the positive and negative aspects, resulted, on the 1–10 VAS, in a mean score of 6.8 (SD = 1.95). Females experienced a higher subjective burden than men (*M*: 6.5 ± 2.0 vs. 7.3 ± 1.8). Results from the different subscales of the COPE index show that 8.6% of the informal caregivers had a score of 16 or more on the negative impact subscale, and on the positive impact subscale 9.5% had a score of 11 or less. About half of the informal caregivers reported at least moderate health problems measured by the EQ-5D for the domains pain/discomfort and anxiety/depression. There was a significant gender difference in perceived mental problems. Female informal caregivers experienced more anxiety/depression than men (*p* = .0008) and also more problems concerning their economic situation (*p* = .02036).

## Discussion

Informal caregivers spent about one half of a full-time working week (40 hours in Sweden) in care-related activities as a consequence of the care recipient’s disorder. Around half of these were used to assist the patient with practical tasks like household work. The other half was reported as ‘stand-by time’ – a care-giving situation that may be specific to psychotic disorders compared to, for instance, patients with Alzheimer’s disease (Max, Webber, & Fox, 1995). In addition to the actual time spent on care giving, the informal caregivers experienced reduced productivity when at work corresponding to a fifth of a full-time working week.

The expenses for informal care giving amounted to an average of €293 per month per informal caregiver, corresponding to 14% of their gross total income. To put this figure into perspective the informal caregivers’ and the care recipients’ mean income was €2153 and €1205, respectively, which is below the mean 2008 gross income of an average Swedish working citizen – €2228 per month (Statistics Sweden, 2008).

The rate of remission (36%) and mean GAF scores (about 50) corresponds with previous findings of remission rates and GAF values in chronic psychosis. Furthermore, the share of patients diagnosed with schizophrenia in outpatient wards for patients with psychotic disorders is similar to that of other studies (Gaite et al., 2005; Placentino et al., 2009; van Os et al., 2006). Thus, the present sample seems to be representative of the population investigated.

The formal care differed substantially from the above-described amount of informal care; a meeting twice a month with a nurse or a contact person, or seeing a doctor four times per year is probably insufficient to meet the needs of the patients and their families. Antipsychotic medication has enabled discharge of patients from mental hospitals but negative and cognitive symptoms, mainly responsible for the functional decline, often remain largely unaltered (Leucht et al., 2009). In most countries, the implementation of evidence-based psychosocial interventions has not accompanied the shift from in- to outpatient care, probably resulting in increased family burden (Dixon et al., 2010; Lehman & Steinwachs, 2003).

There was a great difference in the valuation of time spent between the recall and the diary method, especially regarding the ‘stand-by time’. The informal caregivers underestimated the time when it was recalled compared to the daily diary method. This underestimation may be attributable to an adjustment to these long-term conditions as a normal routine. The fact that informal caregivers above the age of 64 underestimated the time to a significantly greater extent than those under 64 lends some credence to that interpretation. In a Swedish study of informal care giving to patients with reduced health, it was found that informal caregivers who were retired spent more hours on care giving than those who were still working. However, these retired ‘frequent caregivers’ had a significantly lower quality of life compared to the non-frequent caregivers (Borg & Hallberg, 2006). Thus, informal caregivers who have reached the age of retirement may pay less attention to the time spent on care giving and they may also have reached an acceptance and adjustment of their life situation, but this may be at the expense of their own psychological well-being.

Female informal caregivers underestimated the time spent on care giving to a greater extent than men. They also reported higher subjective burden and more anxiety and depressive symptoms, a finding in accordance with other studies (Awad & Voruganti, 2008). The results regarding both retired and female informal caregivers indicate that underestimating the time spent on care giving may constitute a risk factor for reduced psychological well-being. Since psychological distress was perceived as more troublesome than the objective burden, underestimation of time spent on care giving and its relationships with gender, age and psychological well-being should be given more attention in future research.

There are no previous studies comparing the diary and recall methods in psychoses but the diary method has been considered the golden standard in studies of informal care giving of other disorders, rendering credibility to the method (Carton, Loos, Pacolet, Versieck, & Vlietinck, 2000; van den Berg et al., 2006).

Measurement of the subjective burden of care giving (CarerQol-7D) showed that the caregivers suffered from mental health problems and had relational problems. Female caregivers experienced higher subjective burden, in accordance with other studies (Awad & Voruganti, 2008). The mental problems were perceived as more troublesome than the financial, which is somewhat surprising given the considerable amount of time and money spent. This indicates that adaptation to the situation may be pronounced, as also discussed above.

The study did not include a comparison group but, since the COPE index scale has been used in another study, a comparison was attempted. The Swedish subgroup (*n* = 1000) of informal caregivers to older patients in a European multi-centre study, the EuroFamCare study (Lamura et al., 2006), experienced more support from others than the present caregivers. Furthermore, when compared to a representative sample (*n* = 4950) from the Stockholm County, the present informal caregivers experienced more pain, discomfort, anxiety and depression measured by the EQ-5D (Burstrom, Johannesson, & Diderichsen, 2001). This indicates that caregivers to patients with psychotic disorders have more mental health problems due to their care-giving situation and that they receive insufficient support from others.

A wide range of literature exists of informal caregiver burden in other medical conditions, but there are limited reports on psychotic disorders. Few studies of the total cost of psychotic disorders have included family costs and, for those that have, there is a lack of direct measurement of itemized family costs (Awad & Voruganti, 2008). A couple of studies have attempted to estimate the costs, the former using questionnaires and the latter a ‘top-down’ approach, but methodological problems have restricted their accuracy (Davies & Drummond, 1994; Guest & Cookson, 1999; Lauber, Keller, Eichenberger, & Rossler, 2005). Thus, although interventions that require time and engagement from the informal caregivers are recommended in most guidelines, little is known about the caregivers’ ability to carry out such tasks. Political decisions concerning resource allocations to meet the needs of these patients should therefore take into account both formal and informal care, especially since cooperation with informal caregivers is crucial for a favourable prognosis.

The results of the present study raise questions about those patients without informal caregivers. There is evidence that patients receive more informal than formal care and therefore patients without the support of a family are likely to need more formal care (Ochoa et al., 2003). Suggestions for future research are therefore differences in health care consumption between patients with and without access to informal care. Furthermore, the main determinants of the objective and subjective informal care burden and effects of treatment interventions on family burden are also suggested areas for research. A Danish study reported positive effects on family burden by integrated treatment strategies for first-episode psychoses, but there is a lack of studies for those with chronic psychoses (Jeppesen et al., 2005).

To sum up, the informal care-related costs of psychoses has previously not been accurately studied (Awad & Voruganti, 2008). Thus, there is currently very little reliable knowledge regarding the economic and societal consequences of psychotic disorders for families; a surprising fact since interventions involving the family are considered prerequisites for a favourable outcome (Pharoah et al., 2010). The lack of accuracy of previous methods may be one reason that politicians and decision makers do not show enough interest in the costs of informal care giving in psychotic disorders.

## Conclusions

The present study combines a prospective and recall method enabling a comparison that sheds light on important methodological issues. With its bottom-up approach with time and money spent and all the expenditures itemized by a diary method, our study is unique among research on psychotic disorders. The burden of informal care is considerable and its contribution to the care and support of patients with psychotic disorders should be taken into account in health care planning and allocation of resources. Female informal caregivers perceived a higher subjective burden and they also underestimated the time spent on care giving, as did retired caregivers. These gender and age-related risk factors for psychological distress should be further studied and also addressed in clinical practice. The prospective assessment of costs and time spent on informal care giving should be made standard in future studies of the objective burden of care.
